# The Oligomeric Form of the *Escherichia coli* Dps Protein Depends on the Availability of Iron Ions

**DOI:** 10.3390/molecules22111904

**Published:** 2017-11-05

**Authors:** Sergey Antipov, Sergey Turishchev, Yuriy Purtov, Uliana Shvyreva, Alexander Sinelnikov, Yuriy Semov, Elena Preobrazhenskaya, Andrey Berezhnoy, Natalia Shusharina, Natalia Novolokina, Viktor Vakhtel, Valeriy Artyukhov, Olga Ozoline

**Affiliations:** 1 School of Life Sciences, Immanuel Kant Baltic Federal University, 236016 Kaliningrad, Russia; ss.antipov@gmail.com (S.A.); nnshusharina@gmail.com (N.S.); 2 Institute of Cell Biophysics, Russian Academy of Sciences, 142290 Pushchino, Russia; vinnegan@rambler.ru (Y.P.); uliana.shvyreva@gmail.com (U.S.); elena.vl.preobrazhenskaya@gmail.com (E.P.); 3 Department of Biophysics and Biotechnology, Voronezh State University, 394018 Voronezh, Russia; tsu@phys.vsu.ru (S.T.); aa_sinelnikov@mail.ru (A.S.); ysemov@gmail.com (Y.S.); aa.berezhnoy1991@gmail.com (A.B.); novolokina@phys.vsu.ru (N.N.); vakhtel@phys.vsu.ru (V.V.); artyukhov@bio.vsu.ru (V.A.); 4 Department of Cell Biology, Pushchino State Institute of Natural Sciences, 142290 Pushchino, Russia; 5 Department of Structural and Functional genomics, Pushchino Scientific Center, 142290 Pushchino, Russia

**Keywords:** *Escherichia coli*, Dps proteins, oligomerization, ferroxidase, transmission electron microscopy, Mössbauer spectroscopy, molecular docking

## Abstract

The Dps protein of *Escherichia coli*, which combines ferroxidase activity and the ability to bind DNA, is effectively used by bacteria to protect their genomes from damage. Both activities depend on the integrity of this multi-subunit protein, which has an inner cavity for iron oxides; however, the diversity of its oligomeric forms has only been studied fragmentarily. Here, we show that iron ions stabilize the dodecameric form of Dps. This was found by electrophoretic fractionation and size exclusion chromatography, which revealed several oligomers in highly purified protein samples and demonstrated their conversion to dodecamers in the presence of 1 mM Mohr’s salt. The transmission electron microscopy data contradicted the assumption that the stabilizing effect is given by the optimal core size formed in the inner cavity of Dps. The charge state of iron ions was evaluated using Mössbauer spectroscopy, which showed the presence of Fe_3_O_4_, rather than the expected Fe_2_O_3_, in the sample. Assuming that Fe^2+^ can form additional inter-subunit contacts, we modeled the interaction of FeO and Fe_2_O_3_ with Dps, but the binding sites with putative functionality were predicted only for Fe_2_O_3_. The question of how the dodecameric form can be stabilized by ferric oxides is discussed.

## 1. Introduction

The multifunctional Dps protein is best known as the main structural factor of the bacterial nucleoid condensing the DNA of *Escherichia coli* (*E. coli*) upon entering the stage of steady growth [1,2,3,4]. Consisting of twelve identical subunits and possessing a negatively charged surface without typical DNA-binding modules [5], this protein interacts with the bacterial chromosome via twelve unstructured N-terminal tails containing three lysine and one arginine residues [6]. Binding the DNA, Dps protects it from the destructive effects caused by high temperature, electromagnetic and UV radiation, as well as oxidative stress [1]. It is well known that the antioxidant activity of Dps is due to its ability to oxidize Fe^2+^ ions using hydrogen peroxide for this reaction, thereby contributing to a decrease in the effectiveness of the Fenton reaction. Although iron ions are involved in many vital processes, including but not limited to oxygen transport and the creation of functional catalytic centers of many enzymes, the presence of free ferrous ions is toxic to the cell, and is therefore, controlled by a variety of cellular systems. Ferritins, which oxidize ferrous ions and accumulate them in their inner cavity, play an important role in maintaining an optimal balance. Ferritin Dps of *E. coli* has twelve ferroxidase centers located in the subunit contact area [7,8,9]. Each of these has two sites with different affinities for iron ions. The site with a higher affinity consists of His^51^ and His^63^ of one subunit together with Glu^82^ of the adjacent monomer, whereas Lys^48^ and Asp^67^ of one subunit in combination with Asp^78^ of the neighboring monomer form the second site. Since Lys^48^ forms a salt bridge with Asp^67^, the second site is less accessible for interaction with iron ions [7,10]. Although both iron-binding sites are occupied by water molecules in the crystal structure of iron-loaded Dps [7], it is reasonable to expect that in natural environments, at least, the high affinity sites permanently occupy either Fe(II) or Fe(III) in the twelve ferroxidase centers.

Iron ions usually reach the ferroxidase centers through four pores located along the 3-fold axis of the protein globule [11], but their transport can also be carried out through other pores of the protein [9]. The main channels leading to the ferroxidase centers in the Dps-like protein of *Microbacterium arborensis*, are in the form of a truncated cone with a base diameter of about 1 nm and a top of about 0.4 nm with a base oriented outward from the protein globule [12]. The outer surface of these channels consists of negatively charged amino acid residues and additional residues involved in the formation of hydrogen bonds, being conservative for most proteins of the Dps family. The inner surface of the channel contains two or three centers for iron translocation [9], which are closely spaced from each other and contain the amino acid residues of asparagine. Most likely, the force that directs iron ions to the ferroxidase centers of the Dps family proteins, is the charge difference between the inner and outer surfaces of the protein [12,13]. The constant flow of Fe ions through the catalytic centers dynamically affects this difference, modulating the rate of iron accumulation. In the absence of an oxidizer (hydrogen peroxide or oxygen), iron ions remain in the ferroxidase center, creating an excess of positive charge on it, but then they are transferred in a regular flow to the nucleation centers formed in *E. coli* Dps by Glu^64^ and Asp^67^ and enter the process of mineralization.

Thus, even though the ferroxidase centers are only transiently occupied by iron ions, which form electrostatic bonds with the amino acid residues of different monomers, theoretically, increasing the time of their occupancy by increasing the concentration of the substrate can stabilize the oligomeric form of the protein. On the other hand, the integrity of the Dps globule may depend on the size and configuration of the particles formed at the centers of nucleation, and on the filling level of the inner cavity of the protein. The recently discovered dependence of the Dps oligomeric form on the presence of d-glucoronate and d-galacturonate, which causes the dissociation of dodecamers—while the d-glucose did not affect them—indicates a specific dependence of Dps on some low-molecular weight ligands [14]. The aim of this study is to understand whether the natural “internal ligands” of Dps can also affect its structural state.

## 2. Results

### 2.1. Increasing Concentration of Ferrous Ions Stabilizes Dodecameric Form of Dps

**Figure 1:**
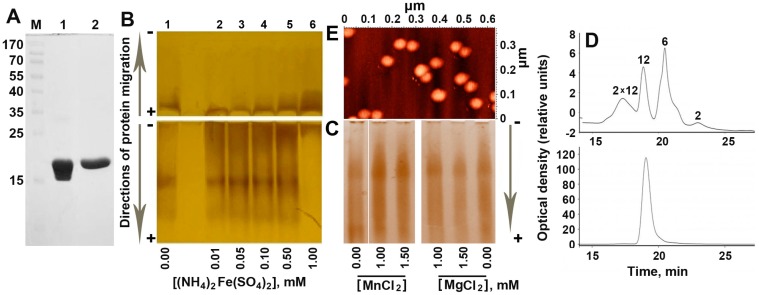
Oligomeric composition of the purified protein Dps. (**A**) The purity level of the protein obtained after ion exchange chromatography (lane 1) and gel filtration (lane 2) as revealed by SDS electrophoresis in 7.5% polyacrylamide gel. Protein marker lengths (M) are indicated in kDa. (**B**) Titration of purified Dps (100 μM calculated for monomers or <8.3 μM for dodecamers) with Mohr’s salt at pH 7.0. Lane 1—native Dps; lanes 2–6—fractional composition of the protein in the presence of 0.01–1 mM of Mohr’s salt, respectively. Vertical arrows on the left indicate the direction of electrophoretic fractionation. (**C**) The same for Dps titrated with MnCl_2_ or MgCl_2_. (**D**) Elution profiles of the Dps from the gel-filtration column in the HPLC system for the purified protein (top panel) and Dps loaded with 1 mM Mohr’s salt (bottom panel). The concentration of Dps used in this experiment was 1 mg/mL (53 μM for monomers). Oligomeric forms corresponding to the observed migration rate are indicated above the peaks. (**E**) Atomic force microscopy (AFM) image demonstrating aggregated Dps particles in the native protein. Since the freshly prepared protein samples required for AFM were fairly homogenous in oligomeric form [18], this method was not used to evaluate effects of Fe(NH4)_2_(SO_4_)_2_.

A specific feature of Dps, which significantly complicates the study of its oligomeric forms and supramolecular complexes, is the propensity for self-aggregation. The same N-terminal tails that participate in the interaction with DNA can also bind the negatively charged surface of other Dps molecules, forming aggregates of a non-standard size [6]. As a result, the efficiency of Dps to form complexes with linear DNA fragments is usually evaluated in electrophoretic mobility shift assays based on a reduced amount of free DNA, rather than on the basis of an increase in the amount of formed complexes [15,16,17,18]. Another technical problem is caused by the positive charge and the variable number of iron ions accumulated in the inner cavity of Dps. The molecular weight of Dps completely saturated with iron ions (~400 ferric oxides per protein globule [8]) is about 15% greater than the molecular weight of the apo protein. As a result, the bands obtained after electrophoretic fractionation with sodium dodecyl sulfate (Figure 1A) differ from the bands obtained under native conditions, when they are always diffuse (Figure 1B,C).

The oligomeric composition of the highly purified protein (Figure 1A) was examined using two methods: electrophoretic fractionation under native conditions (Figure 1B,C) and high-pressure size exclusion chromatography (Figure 1D). Taking into account the contribution of positively charged iron ions to the mobility of Dps oligomers, the samples were electrophoretically fractionated in the forward (from − to +) and in the reverse (from + to −) directions (Figure 1B). To assess the changes in the oligomeric profile, depending on the presence of iron ions, the protein was titrated with a freshly prepared Mohr’s salt Fe(NH4)_2_(SO_4_)_2_, as described in Materials and Methods.

Electrophoretic fractionation (Figure 1B) revealed several oligomeric forms of the protein with different electrophoretic mobility. Two of them had a total negative charge and migrated to the anode. One fraction remained at the top of the gel and the other migrated in the opposite direction, possessing a weak positive charge. The amount of this fraction clearly increased with increasing the concentration of Fe(NH4)_2_(SO_4_)_2_ from 0.1 mM, up to 1 mM, when it became dominant, while small molecular weight oligomers disappeared. The stimulating effect of salts in a high concentration (500 mM NaCl and 2 mM ZnCl_2_) on dodecamer assembling was previously reported for Dps-1 from *Deinococcus radiodurans* [19]. However, in that case, electrophoretic fractionation still revealed the presence of dimers, tetramers and hexamers [19], which were absent when 1 Mm of Mohr’s salt was added to the native protein (lane 6 in Figure 1B). The contribution of ionic strength to the observed effect was tested by the addition of MgCl_2_, or MnCl_2_ instead of Fe(NH4)_2_(SO_4_)_2_ (Figure 1C), but an explicit decrease in the amount of a low molecular weight oligomers was not detected. Ionic strength therefore cannot be considered as the main factor mediating the observed oligomerization. Since the pattern of distribution for the small oligomeric forms detected in the presence of Mn^2+^ (can be oxidized in the Dps ferroxidase centers) was essentially the same as in the presence of Mg^2+^ (is not a Dps substrate), it seems unlikely that the observed oligomerization is mediated by the catalytic activity of Dps.

**Figure 2:**
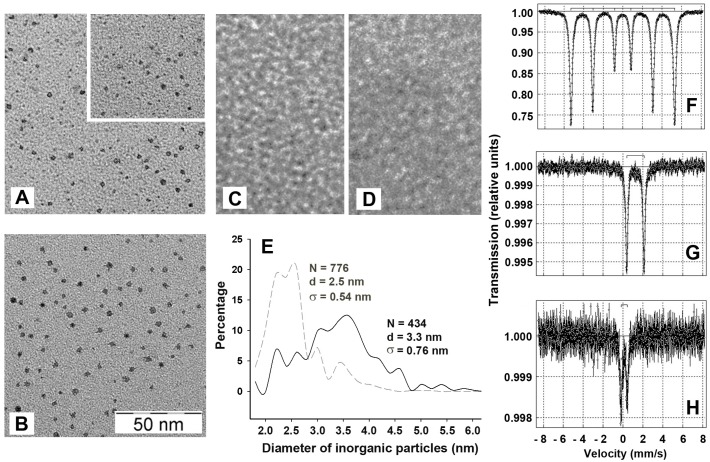
Characteristics of iron content in the inner cavity of Dps. (**A**) and (**B**) TEM images of inorganic particles taken from the native protein (1.64 μM with 126 Fe-ions per dodecamer) and Dps, loaded with 1 mM of Fe(NH4)_2_(SO_4_)_2_, respectively. Particles accounted for image analysis are countered by Altami Studio software (http://altamisoft.ru/products/altami_studio/). An insert in panel A shows an unprocessed image identical to the bottom part of the same panel. (**C**) apo-form of the Dps, prepared as described in Materials and Methods. (**D**) Image of 1 mM of Fe(NH4)_2_(SO_4_)_2_ alone. Scaling is identical for all images and is shown in (**B**). (**E**) Densitograms showing the size distribution of inorganic particles calculated with a resolution step of 0.2 nm. Dashed and solid lines correspond to native and iron-loaded proteins, respectively. **N** denotes the number of analyzed particles, **d**—average diameter of cores approximated by circles, **σ**—StD of **d**. (**F**–**H**) Mössbauer spectra obtained for αFe, 1 mg of dry Fe(NH4)_2_(SO_4_)_2_ and for the Dps protein incubated with 1 mM Fe(NH4)_2_(SO_4_)_2_.

Two dominant oligomeric forms were found in the same sample of the purified protein when the size distribution of multi-subunit complexes was characterized by gel filtration chromatography (Figure 1D, top panel). Based on previously published sedimentation data for the apo-form and the native Dps protein [20], the second peak was attributed to dodecamers (MW ≅ 224 kDa). Peaks of two smaller oligomeric forms correspond to hexamers (MW = 112.17 kDa) and dimers (37.39 kDa). The maximum of the broad peak eluted at ~17.2 min fits to the dimer of Dps dodecamers, which are sometimes found in protein samples. One example of such aggregated particles can be seen in the middle of Figure 1E. As in the case of electrophoretic fractionation (Figure 1B), the addition of 1 mM of Fe(NH4)_2_(SO_4_)_2_ resulted in a dramatic increase in the amount of the high molecular weight oligomer that in the elution profile corresponded to dodecamer (Figure 1D, bottom panel). This effect was well reproducible for Dps preparations with a different pattern of oligomeric forms, including those that possessed dimers of dodecamers. The detected absence of such oligomers in the salt treated sample (Figure 1D, bottom panel) may indicate the surface effect of Fe(NH4)_2_(SO_4_)_2_. Thus, it became clear that ferrous ions stabilize the basic functional oligomeric form of this protein. It is important to note that, despite the conventional point of view that Dps forms stable 12-mers, its oligomeric form depends on several factors and smaller [8,19,21,22] or larger [8] oligomers have also been detected. By changing the pH, for instance, it is possible to get dimers at pH = 5, dodecamers at pH = 7 and again dimers at pH = 8.5 [21]. Moreover, when a sample of dodecamers obtained by gel filtration was again used for fractionation, it contained dimers after only 40 min of incubation [21]. Dodecamers, therefore, tend to dissociate during storage. In AFM experiments, all our protein samples were fairly homogenous, usually containing less than 20% of particles smaller than dodecamers [18]; sedimentation experiments yielded ~10% of putative dimers [20], whereas dynamic light scattering revealed only one peak corresponding to dodecamers [20]. Using freshly prepared Dps, we did not observe small oligomers upon electrophoretic fractionation [14], but detected them with the addition of glucoronate or galacturonate [14]. Once we had found an opposite dependence on iron ions, we used a protein sample that contained both smaller oligomers and larger aggregates to show this. To explain the observed dependence on Fe(NH4)_2_(SO_4_)_2_, we assumed that oligomerization can be caused by the formation of optimal size cores, which play the role of an internal framework for the protein part. On the other hand, a high concentration of iron ions can switch on their ligand-like activity by filling certain binding sites at the interface of Dps monomers inside or outside of the protein globule. Thus, at the next step, we checked the first assumption by comparing the size of the inorganic cores formed in the inner cavity of Fe(NH4)_2_(SO_4_)_2-_treated and native Dps (Figure 2A–E).

### 2.2. The Inorganic Cores in the Inner Cavity of the Dps Protein are Non-Uniform in Size

Transmission electron microscopy (TEM) was used to characterize the size and morphology of internal inorganic cores and its dependence on the level of available iron ions. To achieve saturating levels of iron ions (~450 iron oxides per dodecamer [8,23]) using the same concentration of Mohr’s salt, the protein concentration in these experiments was lowered to 0.4–1.0 mg/mL. Figure 2A,B exemplify the data obtained for the native protein and iron-loaded Dps, respectively. In both cases, the images obtained revealed separated particles distributed over the thin layers of carbon after high vacuum drying. Such particles were practically absent in samples containing apo protein, reconstituted from dissociated subunits as described in Materials and Methods (Figure 2C) and in samples containing only 1 mM of Fe(NH4)_2_(SO_4_)_2_ (Figure 2D). The particles were almost spherical in shape. The analyzing software (Altami Studio) approximated their planar projections as circles. The diameter of these circles was variable in both native and iron-loaded proteins with a maximum measured value of about 6 nm. The particles detected in iron-loaded samples were on average larger (Figure 2B,E), but the overall population remained inhomogenous with even higher level of variability than in the native protein (Figure 2E). This contradicts the assumption explaining the stabilizing effect of iron ions by filling the inner cavity of the protein with the creation of a stable holistic structure.

### 2.3. Mössbauer Spectroscopy Testified the Presence of Fe_3_O_4_ in the Inorganic Content of Dps

It is generally accepted that ferric ions oxidized in ferroxidase centers are stored in the Dps cavity as Fe_2_O_3_ or FeO(OH), i.e., in the form of trivalent ions, while the ferroxidase centers bind Fe(II) ions and convert them in Fe(III) [7,10,24]. Since the ferroxidase centers are only transiently occupied by iron ions, the ratio of Fe(II) and Fe(III) inside the Dps is expected to be very low. On the other hand, the number of iron ions in cells overproducing Dps is limited and cannot saturate all synthesized Dps particles. By decreasing the size of the mineral core this also increases the portion of Fe(II) in the total population of iron ions. In our samples of the recombinant protein, the total level of iron ions varied from ~100 to 250 ions per dodecamer. If Fe(II) ions are located only in bimetallic ferroxidase centers, their percentage should not exceed 10–20%. However, synchrotron experiments conducted by X-ray absorption near edge structure spectroscopy (XANES) showed the presence of more than 50% of ferrous oxides [20]. Despite the fact that the ability of Dps to mineralize Fe^2+^ was previously detected in anaerobic conditions [8], such a large excess over the expected value detected under aerobic conditions was surprising and was tentatively explained by the possible degradation of the protein shell occurring during sample preparation and prolonged drying at room temperature. Therefore, in this study we used Mössbauer spectroscopy for the independent characterization of the charge state of iron ions bound to Dps. The protein concentration for these experiments was maintained at a higher level (2.0–2.5 mg/mL) than in all previous experiments to reduce the Fe^2+^/Dps ratio, thereby increasing the probability of their complete oxidation in the ferroxidase centers of the protein. After incubation with Mohr’s salt, the samples were also passed through a Sephadex G-10 column to remove unbound low molecular weight compound, including Fe^2+^ ions.

The MS1104Em spectrometer was calibrated using the α-Fe standard (Figure 2F), which gave a profile completely consistent with the published spectra (see, for instance, [25]). The spectrum of the dry Mohr’s salt, recorded at room temperature, showed a characteristic doublet, indicating the splitting of the first excited state with some symmetry breaking (Figure 2G).

A reliable Mössbauer spectrum of Dps was obtained only for the iron-loaded sample (Figure 2H). It clearly differs from that of Fe(NH4)_2_(SO_4_)_2_ alone (Figure 2G). The spectrum was processed by the UNIVEM program [26], and was approximated by two quadrupole doublets with isomeric shift (δ) and quadrupole splitting (ΔE_Q_) characteristic for 10 nm spherical nanoparticles of magnetite (Fe_3_O_4_) in the paramagnetic state [27]. Magnetite Fe_3_O_4_ contains both Fe^2+^ and Fe^3+^ ions (mixture of FeO and Fe_2_O_3_ in variable proportions). The Mössbauer spectra of Fe_3_O_4_ nanoparticles for both the paramagnetic (doublets) and for the magnetic state (superposition of sextets), have a non-Lorentzian line shape. Therefore, the best approximation of the Mössbauer spectra for the Dps protein was achieved by the distribution of quadrupole splitting using a non-Lorentz line for two couplet duplets. The parameters obtained after computer fitting are given in Table 1.

The values calculated for quadrupole duplets of the iron-loaded Dps correspond to the Fe^3+^ state of iron atoms in an octahedral environment with two non-equivalent positions, one being more disordered than the other [28]. Taking into account the published data [29] and the value of obtained isomeric shift with respect to the population of *3d 4s* orbitals, the electronic configuration of iron atoms associated with the inner cavity of Dps can be defined as *3d*^5^. Registered spectral parameters best fitted to 50–60% of Fe_2_O_3_ in the total iron moiety of test samples. Thus, the novel data confirmed the presence of bivalent iron in Dps. As 40–50% of the total iron moiety, with a high degree of confidence ascribed to Fe^2+^ cannot be located in the 12 bimetal ferroxidase centers even if the whole content of iron ions has been obtained from Mohr’s salt, at least half of them should be mineralized.

A relatively high spectral similarity of Dps oxides with Fe_3_O_4_ was also observed by XANES spectroscopy [20], though Fe L_3_-edge fine structure registered for Dps sample differed from all the reference spectra including Fe_3_O_4_ nano-powder. The presence of 2t_2g_ and 3e_g_ peaks in the XANES spectra at the soft X-rays absorption edge fine structure unambiguously indicated the presence of trivalent ferric ions in the Dps core. However, the ratio of Fe_3_O_4_ and FeO, estimated by computer simulation of Fe L_2,3_ XANES spectra with the use of reference samples, gave values of 64% and 36%, respectively. The higher percentage of Fe^2+^ ions detected in the analyzed samples by XANES method compared to Mössbauer spectroscopy may be due to essential differences in sample preparation or to some methodological peculiarities of the two techniques. In any case, it became clear that the composition of the Dps inorganic cores has a more complex, non-uniform physical and chemical nature than is usually assumed and to realize or mediate a concentration-dependent protein response to iron ions, in addition to the relatively inert ferric oxides, the Dps protein has soluble and reactive ferrous ions.

### 2.4. Molecular Docking of FeO and Fe_2_O_3_ on the Protein Surface of Dps Revealed a Potential Binding Site for Fe_2_O_3_

**Figure 3:**
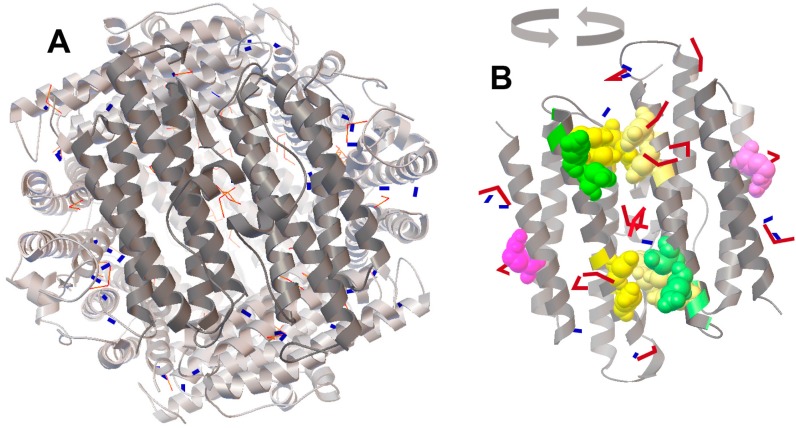
The positioning of FeO (blue rectangles) and Fe_2_O_3_ (red bent models) shown on the surface of dodecamer (**A**) and dimer (**B**). Accession code of the 3D model in the PDB: 1 dps [5]. Locations of Fe_2_O_3_ are accented in (**B**). The docking was performed independently for 50 molecules of FeO and Fe_2_O_3_, and two patterns of positional coordinates were superimposed on the 3D structural model of Dps (left panel). To show the distribution of iron oxides near the ferroxidase centers, two adjacent subunits (dark gray) were rotated for 180° (right panel). Yellow and green spheres show amino acids of ferroxidase and nucleation centers, respectively. Important for the integrity of Dps, R133 [30] is indicated in violet. The 3D structure of the Dps protein was visualized by RasMol 2.7.5 [31].

In the hope of obtaining, at least, some information on how FeO or Fe_2_O_3_ can stabilize the dodecameric form of Dps (Figure 1), we used a method of molecular docking of their structural models on the 3D structure of the Dps dodecamer. We expected to find some additional binding sites for FeO in the interfaces of adjacent subunits, as it is in ferroxidase centers. However, the distribution of 50 sequentially docked molecules turned out to be random (Figure 3A, blue models), except for 12 oxides found close to the ferroxidase centers (Figure 3B). The distribution of binding sites for Fe_2_O_3_ (red models) was more regular and suggested the possibility of their preferential anchoring in the region located between the two catalytic centers (shown in yellow in Figure 3B). These binding sites (the nearest amino acid residues are Ser^44^ and Leu^45^ of one monomer, as well as Arg^70^ and Ile^74^ of another) are close to the nucleation centers (shown in green), but do not overlap with them. Thus, it is possible that a high concentration of iron ions favors an increase in the mineralization area on the inner surface of Dps.

However, of the two amino acid substitutions (W52A and D78A) made at this protein interface near the ferroxidase centers, only W52A slightly decreased the stability of Dps [30]. On the other hand, the substitution R133A located on the opposite site of the monomer bundle (indicated in violet in Figure 3B) and positioned near the ferritin-like three-fold symmetry axes shifted the oligomerization state to ~50% of dimers [30]. The docking performed for the entire Dps globule revealed Fe_2_O_3_ oxides near these residues (Figure 3B). Thus, we assume that ferric oxides occupying different places on the protein surface, including those which are far from ferroxidase centers, can play a specific role in the stabilization of the dodecameric form of Dps.

## 3. Discussion

Besides ferroxidase activity and the ability to condense bacterial DNA in stress conditions, Dps is involved in several other cellular processes. Proteins of this family have been found among components of the outer membrane [32] and participate in biofilm formation [33]. However, the protective role of Dps, mediated by its ability to scavenge toxic ferrous ions and to remodel genomes into a heterochromatin state [2,3,4,6], is without a doubt, biologically much more significant. Oxidative stress and starvation induce the most serious Dps-mediated rearrangements in bacterial DNA, which is converted in tightly packed toroid-like or even crystal-like structures [34,35,36,37,38]. By protecting the genome from various harmful compounds, this condensation also suppresses transcriptional activity in the cell, which must be easily restored after exiting stress or starvation. That is why, it was reasonable to assume that the DNA-binding capacity of Dps can be modulated by components with a physiologically dependent presence in bacterial cells. Recently, two such ligands from the metabolic pathway, which are more energetically effective than glycolysis (d-glucuronate and d-galacturonate) have been found [14]. By destabilizing the dodecameric form of Dps, they provide the bacteria with a mechanism that, in response to the appearance of nutrients, can stop Dps-mediated condensation of the genome, and consequently, restore its lost functionality.

Iron has the same critical importance for the growth of bacteria as a source of nutrition, and its concentration in cells strongly depends on cell density [39]. Therefore, it is not surprising that iron ions affect the structure of the protein, which naturally interacts with them and plays a decisive role in the protective packaging of the bacterial chromosome. Data from both electrophoretic fractionation and gel filtration experiments are extremely convincing (Figure 1B,D), but the most important observation made in this study is the opposite effect of iron ions on the oligomeric state of Dps compared to hexuronates [14]. Since during the fasting, when almost the entire genome is covered with Dps [2,3,4], the concentration of iron ions in the bacterial cells is lower than in the rapidly growing culture [39], whereas the number of Dps molecules is ~15–30-fold greater [2,3,4], it is clear that the Dps particles cannot be saturated with iron and should be relatively unstable. This is an important factor that allows for the maintenance of condensed chromatin in the conformation available for rapid functional recovery. On the other hand, the assembly of apo protein in dodecamers can be easily achieved in vitro [20,40]. The presence of iron ions is therefore not obligatory for oligomerization ensuring the ability of Dps to package genomes even in iron-depleted conditions and adapt the level of condensation to growth conditions.

The question of how an excess of iron ions can affect the oligomeric form of Dps remained mostly unresolved. This could be explained by additional inter-subunit sites for Fe^2+^ binding, but model experiments did not find these. However, we revealed a potential site for nucleation of Fe_2_O_3_ located nearby catalytic centers that can be involved in mineralization, while ferric oxides bound to the ferritin-like vehicles potentially can act as structure stabilizing ligands. We also found that the size of the inorganic cores of Dps is variable both in purified protein (Figure 2A,E) and in Dps loaded with additional iron ions (Figure 2B,E). It is likely that the difference in size reflects the difference in the time of biosynthesis. In those protein particles that were formed earlier and began to accumulate iron ions, inner cores could be larger and grow faster simply because of the larger surface of mineralization. The peak maxima in size distribution observed in different experiments with iron-loaded protein varied from 2.8–3.5 nm, which is smaller than the diameter of the inner cavity—4.5–5 nm [5]. However, 2–3% of detected inorganic particles were larger than 5 nm. Perhaps, in dodecamers maximally filled with iron oxides, the mineral core includes clusters located in the centers of nucleation, i.e., there is partial penetration into the inter-subunit space.

The 3D structure of Dps assumes that the positively charged N-tails are grouped in triads [5]. That is why the segments of branched DNA are more energetically preferable for binding [18]; they provide more platforms for interaction. However, in this case, the genetic material appeared in the immediate vicinity of the pore for ferrous ions migrating to the ferroxidase centers or in the opposite direction, if they are required for cellular metabolism. This means that a dangerous combination of H_2_O_2_ and Fe^2+^ appears in close proximity to genomic material. In this paper, using Mössbauer spectroscopy, we confirmed the presence of ferrous ions, not only in the ferroxidase centers, but also in the mineral core. Although the ability of Dps to accumulate ferrous ions was reported at the very beginning of its study [8], this is often ignored; but it should be taken into account due to the multifunctional nature of this protein.

## 4. Materials and Methods

### 4.1. Isolation and Purification of Dps Protein

Isolation and purification of the Dps protein was carried out according to a previously developed procedure [17] from *E. coli* BL21* cells transformed with PGEM_*dps* plasmid. In the final step it was dialyzed against buffer containing 50 mM Tris-HCl (pH 8.0), 50 mM NaCl and 10^−4^ M EDTA, frozen and stored frozen until use. The concentration was measured using a ND-1000 spectrophotometer (NanoDrop Technologies Inc., Wilmington, DE, USA). All protein preparations were tested for iron content using a 1.10-phenantroline method. Depending on the level of over-production, it varied from 100 to 250 Fe-ions per dodecamer. Under condition of strong induction, the iron stock in the medium is rapidly depleted, causing a higher heterogeneity. Therefore, we used a very low concentration of IPTG (0.02 mM) to make induction as delicate as possible. To determine the total iron content by the 1,10-phenantroline method, 5 μL of 10% hydrochloric acid and 10 μL of ~40% sodium acetate buffer (3 M CH_3_COONa + CH_3_COOH so as pH≅4.0–5.0) were added to 25–50 μL of the protein solution. Samples were boiled for 5 min and transferred onto a plate with 5 μL of 0.1% 1,10-phenanthroline. The volume was adjusted to 250 μL with distilled water, and the probes were incubated at room temperature for 15 min. Absorbance was measured with a Multiskan PLUS spectrophotometer (Labsystems, Vantaa, Finland) at 492 nm. The number of iron ions per Dps particle was calculated using a calibration plot built on a series of dilutions of FeCl_3_ and/or Mohr’s salt. To obtain the apo-form, the purified Dps was first dialyzed against a buffer containing 10 mM Tris-HCl (pH 8.0), 50 mM NaCl and 5% glycerol. Then, the iron content was removed from the protein globule by dialysis in a pH gradient 8.0 → 7.0 → 6.0 → 5.0 → 3.8, and dodecamer structure was restored by further dialysis: 3.8 → 5.0 → 6.0 → 7.0 → 8.0. Each step took 1–2 h. The quality of the obtained apo protein is shown in [19].

### 4.2. Electrophoretic Fractionation

To load the purified protein with additional iron ions, it was dissolved in a buffer, containing 50 mM Tris-HCl (pH 8.0), 200 mM NaCl and 10^−4^M EDTA, and mixed with a solution of freshly prepared Fe_2_(NH_2_)_2_(SO_4_)_2_·6H_2_O (Mohr salt), MgCl_2_ or MnO_2_. Samples (20 μL) containing 100 μM Dps monomers (<8.3 μM of dodecamers) were incubated for 30 min at room temperature with increasing salt concentration and loaded on a 5% polyacrylamide gel for electrophoretic fractionation under native conditions. The electrophoresis buffer contained 89 mM Tris-HCl (pH 8.0), 89 mM boric acid, 2 mM EDTA. Fractionations in forward (from − to +) and reverse (from + to −) directions were conducted in parallel using an electric field with a voltage of 200–250 V and a current strength of 70–110 mA. The bands were visualized by staining the gel with AgNO_3_.

### 4.3. Size Exclusion Chromatography

The patterns of oligomeric forms in the highly purified protein and in the iron-loaded sample were assessed by using the HPLC system equipped with a UV detector (Marksk, Hitachi, Germany) and a 300 mm sephacryl-200 column with a diameter of 15 mm (Amersham, Biosciences, Buckinghamshire, UK). The column was pre-equilibrated with a buffer containing 50 mM Tris-HCl (pH 7.0), 0.1 mM EDTA, 0.1 mM DTT, 0.2 M NaCl and 0.6 M urea. Eighty microliters of protein solution (1 mg/mL or less than 4.4 mM of dodecamers) was incubated for 30 min at room temperature in the same buffer with or without 1 mM of freshly prepared Fe_2_(NH_2_)_2_(SO_4_)_2_·6H_2_O and was applied to the column. Elution was performed with a flow rate of 1 mL/min.

### 4.4. Atomic Force Microscopy

Sample preparation for atomic force microscopy was performed as described in [18]. This included filtration of protein samples through Sephadex G-15 column (1 × 5 cms) and dilution in the buffer containing 50 mM Tris-HCl (pH 7.5) with 10 mM NaCl, to the final concentration of 1 ng/μL (<4.4 nM of dodecamers). Two microliters of this solution were loaded on mica and held there for 5 min. Then, the sample was washed, dried, and scanned by AFM Integra-Vita (NT-MDT, Zelenograd Russia).

### 4.5. Transmission Electron Microscopy

Thin carbon layers were wetted with a protein solution containing purified Dps or iron loaded protein at a concentration of 0.4–1.0 mg/mL and vacuum dried within a camera of the microscope. Measurements were performed with the use of the Carl Zeiss Libra 120 microscope. The accelerating voltage was 120 kV. Obtained images were analyzed using Altami studio 3.4 software (http://altamisoft.ru/products/altami_studio/, Altami Software, St. Petersburg, Russia). The procedure included background correction, identification of inorganic particles and their characterization. The detected particles were approximated by circles whose diameter was used as a measure of the size. To eliminate the noise given by technical impurities, only spherical particles with a coefficient of circularity in the range from 1.0 to 1.2 were counted.

### 4.6. Registration of Mössbauer Spectra

Iron-loaded Dps samples were prepared as described above from 2.0–2.5 mg/mL protein solutions. Unbound iron ions were removed by filtration through Sephadex-G10 (Pharmacia, Sweden). A cuvette with a sample in a volume of 150–200 μL was frozen to a nitrogen temperature and placed on the movable rod of the modulator. The Mössbauer spectra of samples frozen up to 77 °K were recorded using a spectrometer MS1104Em (Department of Physics, Southern Federal University, Rostov-on-Don, Russia) using absorption geometry and in the mode of constant accelerations (±8 mm/s) at 77 °K. The spectrometer analyzer recorded the spectra in 1024 channels. The activity of the source at the time of measurement was 20 mCi. To calibrate the spectrometer, a reference sample of metallic iron α-Fe was used. Mössbauer spectra were processed using the UNIVEW software and χ^2^ test.

### 4.7. Molecular Docking

To model the interaction of FeO and Fe_2_O_3_ with the oligomer of the Dps protein, a method of sequential molecular docking was used and it was carried out essentially as described in [14]. The 3D model of the Dps dodecamer obtained by Grant et al. [5] was taken from the Protein Data Bank (code: 1 dps). Molecules of water and ions of sodium were removed from the published structure. Models of FeO and Fe_2_O_3_ were obtained using Avogadro, version 1.2.0n-win32 (https://avogadro.cc, University of Pittsburgh, Pitsburg, PA. USA [41]). Their geometry was optimized by means of 15 iterations in a universal force field. The location of the oxides on the Dps surfaces was estimated using a software package Autodosk VINA [42]. Consecutive computations were independently performed for 50 sequential steps of docking for FeO and Fe_2_O_3_. At each step, 10 sites with the highest affinity were obtained and one with the largest value was added to the target model. This new model was then used as a target for the next round of docking. As a result, the two final models contained one molecule of the dodecamer DPS and 50 molecules of FeO or Fe_2_O_3_.

## Figures and Tables

**Table 1:** The values of hyperfine spectrum parameters for Mohr salt and DPS protein.

Sample	Approximation	δ, mm/s	ε, mm/s	G, mm/s	S, %
α-Fe	Sexstet_1	0			
Mohr salt	Doublet_1	1.39			
Dps with Mohr salt	Doublet_1	0.25 ± 0.02	0.60	0.22	37
	Doublet_2	0.27 ± 0.02	0.62	0.22	63

δ—isomeric shift, ε—quadrupole splitting, G—widths of lines at half-height, S—the area of an individual subspectrum as a percentage of the total spectral area.
